# Full Optimization of an Electroless Nickel Solution: Boosting the Performance of Low-Phosphorous Coatings

**DOI:** 10.3390/ma14061501

**Published:** 2021-03-18

**Authors:** Asier Salicio-Paz, Ixone Ugarte, Jordi Sort, Eva Pellicer, Eva García-Lecina

**Affiliations:** 1 CIDETEC, Basque Research and Technology Alliance (BRTA), E-20014 Donostia-San Sebastián, Spain; asalicio@cidetec.es (A.S.-P.); iugarte@cidetec.es (I.U.); 2 Departament de Física, Universitat Autònoma de Barcelona, E-08193 Bellaterra, Spain; jordi.sort@uab.cat (J.S.); eva.pellicer@uab.cat (E.P.); 3 Institució Catalana de Recerca i Estudis Avançats (ICREA), E-08180 Barcelona, Spain

**Keywords:** electroless nickel, Taguchi, optimization, hardness, wear, corrosion

## Abstract

Univariate and multivariate optimizations of a novel electroless nickel formulation have been carried out by means of the Taguchi method. From the compositional point of view, adjustment of the complexing agent concentration in solution is crucial for fine-tuning free Ni^2+^ ions concentration and, in turn, the mechanical properties of the resulting coatings. The Ni (II) concentration and the pH are the main parameters which help restrict the incorporation of phosphorous into the Ni layers. On the other hand, the stirring rate, the pH and the reducing agent concentration are the most influential parameters for the corrosion resistance of the coatings. Multivariate optimization of the electrolyte leads to a set of optimized parameters in which the mechanical properties (hardness and worn volume) of the layers are similar to the optimal values achieved in the univariate optimization, but the corrosion rate is decreased by one order of magnitude.

## 1. Introduction

Since the discovery of electroless nickel plating (ENP) by Brenner and Riddell in 1946 [1], this formulation has become one of the preferred solutions for engineering applications in which high corrosion resistance and superior mechanical properties are required [2]. In essence, and leaving aside the mechanistic phenomenon behind the electroless formation of NiP, which still remains unsolved [3,4], the electroless plating method is an autocatalytic metal deposition in which the reduction in metallic cations is driven by a reducer, present in the same solution, without the use of external power supply [5].

ENP electrolytes are multicomponent electrolytes typically containing a source of nickel cations, a reducer and several complexing agents, in addition to stabilizers, buffers, accelerators and wetting agents [6]. All these components fulfil essential functions during the electroless process and their careful selection, along with the fine tuning of the experimental conditions, have a pronounced effect on the properties of the resulting coatings [7].

The properties of electroless nickel coatings are mainly governed by the phosphorous (P) content in the alloy [8]. Low phosphorous deposits (3–5 wt.% P) exhibit superior mechanical properties but poor corrosion resistance in acid media; medium phosphorous coatings (6–9 wt.% P) show moderate corrosion resistance and good mechanical properties and, finally, high phosphorous layers (10–12 wt.% P) exhibit superior corrosion resistance at the expense of limited mechanical performance [9,10,11,12,13]. In addition to the phosphorous content, careful choice of bath composition, operating pH and temperature of the system have an effect on the properties of ENP layers [14].

In this work, the best mix of bath composition (i.e., components and their concentration) and operational parameters were targeted in order to optimize a proprietary electroless nickel formulation. As a result of the large number of factors to be considered, the Taguchi method was used to elucidate the effect each factor had on the performance of the bath and the properties of the resulting coatings. The Taguchi method consists of three phases; namely system design, parameter design, and tolerance design [15]. System design involves the selection of the optimal factor levels for developing the quality characteristics. In this research, system design was utilized in combination with multivariable analysis to determine the optimum set of bath components, concentration and working parameters which yielded the best corrosion resistance, mechanical and tribological properties of the coatings.

In contrast to full factorial analysis, the Taguchi method reduces the number of experimental runs by using orthogonal arrays (OAs) in which all the factors and their levels are included, defining the experimental setup during the research [16]. In our study, an L_8_ OA was utilized to explore the effect of the most influential parameters of the new ENP bath, where the letter L in this notation stands for Latin square. The concentrations of the nickel salt (A), the reducing agent (B), the complexing agents 1 (C) and 2 (D), the stirring rate (E), the temperature (F) and the pH (G) were selected as the most influential parameters. Additionally, two levels were set for each factor, depicted by “−” for the lowest and “+” for the highest levels, respectively, completing the L_8_ OA as shown in Table 1. The effect of process parameters and bath composition on the thickness, hardness, corrosion resistance and wear of the coatings was evaluated separately based on individual optimization stages for each feature. Nonetheless, coatings working under real-life conditions are usually subject to more than one degradation mechanism (i.e., corrosion, wear, fretting, etc.) simultaneously. For this reason, a multivariate analysis was carried out aimed at gathering the conditions leading to both best wear and corrosion resistance for the here considered low phosphorous electroless nickel coatings.

## 2. Materials and Methods

### 2.1. Electroless Nickel Bath and Operating Conditions

A proprietary low P electrolyte has been used in this study. Main components of the solution include NiSO_4_ as Ni (II) cations source, sodium hypophosphite as reducing agent, short chained organic acids as complexing agents (i.e., malic acid, glycine, citric acid, propionic acid, etc.) and ammonia and diluted sulfuric acid as pH modifiers. Apart from bath compositional factors, some working parameters, namely solution pH, temperature and stirring rate were included as parameters to be optimized.

All the reagents were of analytical grade and were dissolved in distilled water. Note that the quality of reagents is important in electroless nickel formulations as these electrolytes are very sensitive to metallic impurities, usually present in low purity chemicals. Importantly, the ENP formulation used here meets the requirements of the RoHS and WEE standards [17]. Coatings were plated in 1L cylindrical glass cells for a total plating time of 1 h. Four NiP coatings were manufactured per condition during the optimization stage. Temperature of the electrolyte was kept fixed by means of a PT-1000 probe attached to a hot plate magnetic stirrer (RTC basic, IKA, Staufen im Breisgau, Germany). The probe ensured a maximum temperature deviation of ± 1 °C. The electrolyte was stirred with a Teflon^®^-coated magnetic bar (Wilmington, NC, USA) placed at the bottom of the cell. Meanwhile, a polyethylene cover was placed on top of it to prevent from water evaporation and consequent changes in the concentration of the species in the electrolyte, and to maintain a stable temperature across the cell.

### 2.2. Substrate Conditioning

The substrates were low carbon steel (AISI 1010) [18] flat panels (100 mm × 75 mm × 1 mm). In order to assure good adhesion between the substrate and the electroless nickel coatings, the former was subject to surface conditioning. Firstly, after soak cleaning, the substrates underwent chemical degreasing (UniClean 251, Atotech Deutsland GmBH, Berlin, Germany) for 5 min at 60 °C followed by electrolytic degreasing at 4 V for 1 min in the same solution. A stainless-steel anode was employed during the electrolytic degreasing. The substrates were then subject to acid pickling for removing the outermost oxide layer from their surface. The acid solution was composed of HCl and H_2_SO_4_ at 40% and 5% (*v*/*v*), respectively. Once gently rinsed, the substrates were transferred to the plating cell.

### 2.3. Selected Orthogonal Array

The parameters subject to optimization were the concentrations of nickel cations (A), reducing agent (B) and complexing agents 1 (C) and 2 (D), the stirring rate (E), the temperature (F) and the solution pH (G). In the Taguchi approach, the degrees of freedom (DoF) of a system are defined as DoF = *number of factors* − 1. In our case, as there were seven parameters to explore, the system had six DoF. Thus, the smallest OA able to allocate six DoFs is an L_8_ (2^7^) matrix, which enables the study of seven factors at two levels, where “−” and “+” in the OA stand for lower and upper levels, respectively, for each factor. The different factors and their levels are distributed in the resulting matrix, giving rise to the sequence of experiments to be done. Each row defines the experimental setup for every single test.

Careful positioning of the factors in the L_8_ OA needs to be done beforehand in order to anticipate possible interactions between factors [19]. An interaction between two factors occurs when the effect of a factor on the considered feature or property depends on the level at which the other factor is operating, thereby hiding or polluting the real effect of the former. As the OA was populated with seven factors, these were placed in matrix positions for which, according to common knowledge in electroless plating, interactions between parameters are not expected. The final experimental matrix derived from the OA is shown in Table 1.

The Taguchi methodology involves a graphical representation of the parameters, which further facilitates detection of the most influential ones on the properties to boost. The results are converted into signal-to-noise (S/N) ratio according to three main categories, namely *lower-the-better, higher-the-better,* and *nominal-the-best*. The objective of the optimization stage is to maximize the S/N ratio in order to achieve the best quality characteristics. According to the methodology, it is possible to predict the response of each feature according to the optimal set of parameters derived from the optimization stage. The prediction is then validated in a confirmation test or experiment [20].

Thus, in order to unveil the effect that the different bath components and the working parameters (pH, stirring rate, and temperature) have on the properties of the resulting coatings, thickness, hardness, wear and corrosion resistance were selected as the outputs of individual univariate optimizations. On a further step, a multivariate optimization was carried out for all the features mentioned above, by prioritizing corrosion and wear resistance as the properties to be improved.

### 2.4. Characterization of the Coatings

Surface topography and thickness analyses were carried out on a Jeol JSM 5500LV scanning electron microscope (SEM, Tokyo, Japan). Compositional analyses of the samples were performed in the SEM by means of an Oxford instruments INCA X-sight X-ray spectroscopy (EDX, Abingdon, UK) detector. In some cases, coatings were analyzed on a Zeiss Gemini field emission scanning electron microscope (FESEM, Oberkochen, Germany) equipped with an Ametex EDAX Apollo X detector. Thickness and composition of the coatings were double checked by means of Fischer Instruments XDV-SDD X-ray fluorescence instrument (XRF). Cross-sections of the coatings were prepared by cutting and embedding in hot mounting epoxy resin, grinded with SiC paper (1400–4000 grit) and polished down to a mirror-like finishing using diamond paste (1 µm). Crystalline structure of the coatings was analyzed on a Bruker D8 X-ray diffractometer(Billerica, MA, USA) using CuKα radiation in Bragg Brentano geometry. Phase analysis was done using Bruker EVA^®^ software package [21]. Crystallite size was evaluated using the Scherrer’s formula. 

Hardness of the coatings was assessed with the help of a Fischer Instruments Fischerscope HM 2000 (Fischer Instrumentation(GB)Ltd., Lymington, UK), using a pyramidal-shaped diamond Vickers indenter, by applying a normal load of 100 mN. The obtained values correspond to the average of 10 indentations performed on the cross-section of the coatings. Wear performance of the coatings was evaluated using a CSM THT tribometer in ball-on-disc configuration (CSM Instruments, Needham, MA, USA). The tribo-pair consisted of an Al_2_O_3_ ball (Ø = 6 mm) in contact with the NiP coatings. The applied normal load was 10 N during a total sliding distance of 3000 m at a rotating speed of 10 mm/s. The wear tracks were evaluated by a Leica Microsystems DCM3D (Leica Microsystems, Wetzlar, Germany) confocal microscope, operated with a blue light (λ = 460 nm) at a 10× magnification by means of a 10× EPI objective. The total worn volume was calculated using LeicaMaps (Leica Microsystems) dedicated software.

Corrosion performance of the coatings was studied by electrochemical techniques. Experiments were conducted on a Princeton Applied Research cylindrical cell with a total volume of 250 cm^3^ and an exposed testing area of 1 cm^2^. The electrolyte was NaCl 3.5 wt.% (Scharlau, ACS), a Crison 52–41 Ag/AgCl/KCl (3M, St. Paul, MN, USA) was employed as a reference electrode whereas a platinized titanium mesh was used as a counter-electrode. All measurements were conducted under stagnant conditions at room temperature. During the experiments, open-circuit potential (OCP) values were recorded for 1 h in order to ensure a stable value before running the polarization curves. Separate cathodic and anodic potentiodynamic curves were recorded in the range of (−0.050/+1.0) V vs. OCP and (+0.050/−1.0) V vs. OCP, respectively. Corrosion rate was extrapolated from the analysis of the corresponding Tafel slopes.

## 3. Results and Discussion

### 3.1. Univariate Optimization

#### 3.1.1. Composition and Crystallographic Structure

In the pursuit of achieving the best mechanical properties, the lowest possible P content was targeted. Therefore, the lower-the-better approach was utilized to find the optimal combination of factors leading to the lowest P content. Note that the S/N ratio is determined differently depending on the type of characteristic being analyzed [22].

As can be seen in Table 2, the phosphorous content varies in the range from 2.2 to 8.5 wt.% P as a function of both bath components concentrations and working parameters.

**Figure 1:**
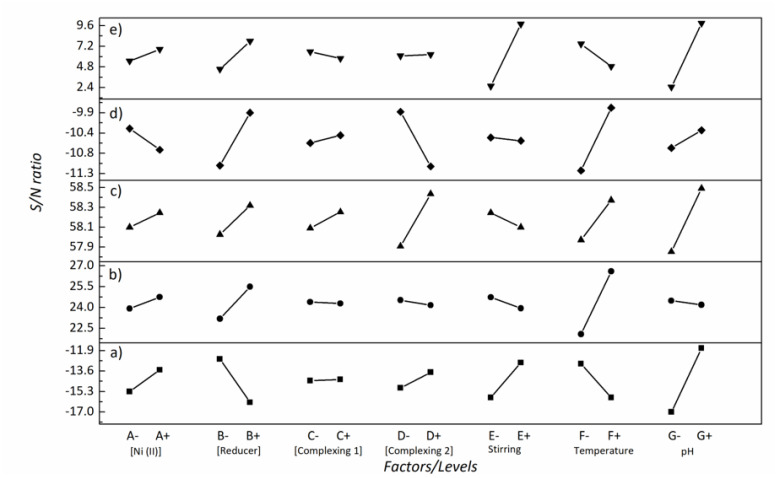
Signal-to-noise (S/N) ratios for (**a**) P content, (**b**) thickness, (**c**) hardness, (**d**) wear resistance (worn volume), and (**e**) corrosion resistance (corrosion current) of electroless nickel plating (ENP) layers for the differently analyzed factors and their levels (−, +).

Figure 1a shows that the pH value (G) and the concentration of reducing agent (B) are decisive in achieving the lowest P content. Meanwhile, stirring and temperature also have an effect, although lesser. This can also be observed in the mean response values based on S/N calculations (Table 3).

Taking into account the data displayed in Table 3, next step in the Taguchi’s methodology consists in solving a prediction equation in which a projection of the P content is obtained according to the optimum parameters sequence. In our case, the optimal combination of factors towards the lowest P content was A+B−C+D+E+F−G+, forecasting a value of 3.3 wt.% P. A confirmation experiment was run at the optimized conditions and the resulting P content was 3.1 ± 0.2 wt.%, in full agreement with the prediction. 

Remarkably, the profound effect of pH on the P content in electroless nickel coatings has been described elsewhere [23]. On the contrary, the reducing agent concentration is not typically considered a key factor in the literature [7]. As can be seen in Figure 1a, lowering the reducing agent concentration brings a decrease in the P content (maximum S/N). Likewise, an increase in the Ni (II) concentration in solution decreases the P content in the layer. These results confirm the importance of the Ni (II)/H_2_PO_2_^−^ ratio in solution, so that the higher the ratio within a certain range, the lower the P content [7].

**Figure 2:**
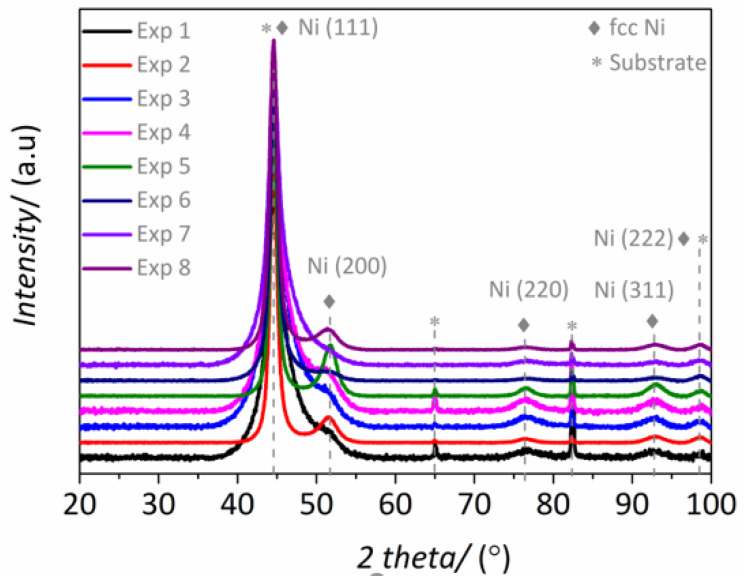
XRD pattern of the NiP coatings obtained from Experiments 1–8 as listed in Table 1.

The crystalline structure of NiP coatings is also strongly dependent on the P content. Figure 2 shows the XRD patterns of the eight samples derived from the experimental matrix. All the peaks can be indexed considering the face-centered cubic (fcc) phase of Ni, the (111) being the preferred orientation in all cases. Besides the (111) reflection, the patterns related to experiments 2, 5 and 8 show a sharper and better resolved (200) peak, indicating a higher degree of crystallinity. Accordingly, coatings derived from these experiments had the lowest P contents, namely 3.2, 2.5 and 4.0 wt.%, respectively. Because phosphorous has very low solubility in nickel, the Ni lattice becomes severely distorted when both elements are co-deposited [24]. The broadening of the (111) peak in the XRD patterns of coatings having higher P percentages results from the increased lattice distortion caused by the incorporation of P in the Ni matrix [25]. Note that P tends to accommodate at the grain boundaries, thus inhibiting grain growth, which ultimately causes the observed peak broadening (i.e., smaller crystallite sizes) [26].

#### 3.1.2. Thickness and Surface Morphology

In this study, maximum thickness of the ENP layers was pursued during the optimization stage. Unlike P content, the optimization of thickness fell within the higher-the-better category. Figure 1b shows the main effects on thickness, for which temperature and reducing agent concentration emerged as the most influential parameters. Higher concentrations of both nickel cations and reducing agent caused an increase in thickness, in agreement with the empirical rate law for auto-catalytic deposition [27]. Thus, the best configuration of parameters to achieve the maximum thickness was A+B+C−D−E−F+G−. The predicted thickness value was 24.1 µm, very close to the one obtained in the confirmation experiment (24.3 ± 1.6 µm), thereby confirming that the chosen configuration of parameters was indeed optimal.

**Figure 3:**
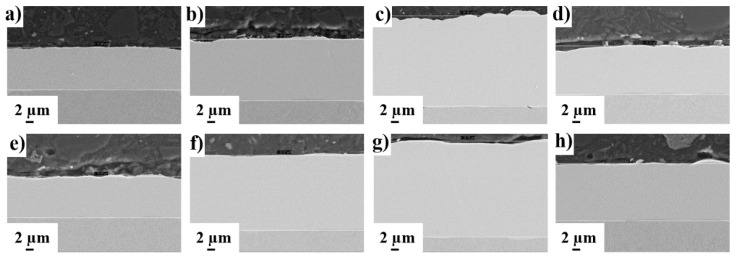
SEM images of the cross-section of ENP coatings obtained from experiment number (**a**) 1, (**b**) 2, (**c**) 3, (**d**) 4, (**e**) 5, (**f**) 6, (**g**) 7 and (**h**) 8.

Temperature is the driving force in electroless plating processes and its influence on coating thickness can be seen in Figure 1b. For completeness, Figure 3 shows the SEM images of the coatings’ cross-sections for the different studied conditions. The results indicate that experiments 2, 3, 6 and 7 yielded the thickest coatings. In these experiments, the temperature was set in its upper level (90 °C), pointing to a higher effect of temperature on thickness than the other parameters. Theoretically, experiments 2 and 3 were the most favorable conditions toward thicker coatings. However, in the case of experiment 2, lower Ni (II) concentration and higher concentration of complexing agent 2 (see Table 1) decreased the amount of free Ni^2+^ cations available for reduction by H_2_PO_2_^−^. As a result, this experimental configuration reduces the effect of temperature on the plating rate, although it is still higher in comparison with experiments operating at lower temperatures. Meanwhile, experiments 6 and 7 were conducted with the temperature factor set at the upper level (90 °C) and the pH at the lower level (5.2) (Table 1). The coating derived from experiment 7 was the thickest. Importantly, in this case the concentrations of both Ni (II) and H_2_PO_2_^−^ were in the upper range, whereas the concentration of complexing agents 1 and 2 were in the lower range, thus setting a scenario in which the amount of free Ni^2+^ cations to be reduced is higher.

**Figure 4:**
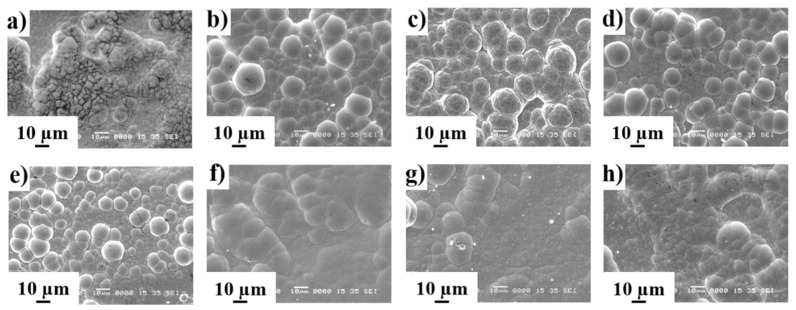
Top-view SEM images of ENP coatings obtained from experiment number (**a**) 1, (**b**) 2, (**c**) 3, (**d**) 4, (**e**) 5, (**f**) 6, (**g**) 7 and (**h**) 8.

Bath components concentrations and working parameters also have an effect on surface topography of coatings as can be seen in Figure 4. All the coatings showed the typical nodular growth with cauliflower-like endings randomly distributed on the surface showing differences in size and nodules distribution. Among the different factors able to impact surface topography, pH and the nature and concentration of complexing agents are the most important ones and, presumably, they act together (i.e., mixed effect) on surface topography of electroless nickel coatings [28,29,30].

#### 3.1.3. Hardness

**Figure 5:**
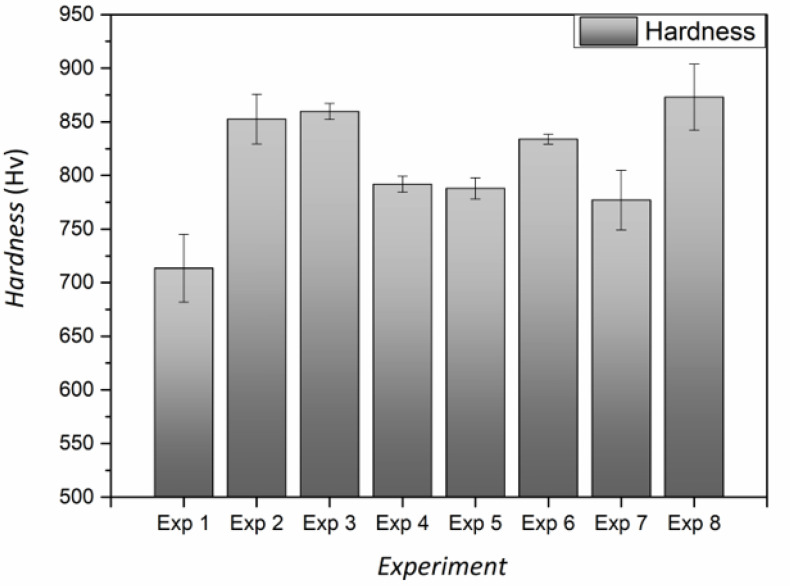
Hardness values of electroless nickel coatings obtained from experiments 1–8.

As can be seen in Figure 5, the hardness of the coatings, obtained from the different experimental conditions, shows that experiments 2, 3, 6 and 8 led to the highest coating hardness values, with a top value of 873 ± 31 Hv_0.1_. These values were near those typically exhibited by hard chromium coatings (HCC) obtained by both direct current and pulse plating, which lie in the range of 1000–1100 Hv, and also near the hardness of some HCC substitutes like electrodeposited Ni-W alloys [31,32]. These results confirmed the preeminent position of electroless nickel coatings as a greener alternative to HCC coatings in terms of hardness. This fact along with the main benefits of electroless plating technology (i.e., thickness homogeneity, low concentration of metallic ions, tunable properties depending on P content, no need to apply an external electric field, etc.) make ENP one of the most promising alternatives to HCC for functional applications.

Figure 1c indicates that the most influential parameters to secure high hardness were pH and concentration of complexing agent 2, followed by temperature and reducing agent concentration to a lesser extent. The optimum configuration of parameters to maximize hardness of coatings was A+B+C+D+E−F+G+. According to this parametric setup, the prediction equation yielded a hardness value of 870 Hv_0.1_. Coatings fabricated in the confirmation experiment had a hardness of 863 ± 27 Hv_0.1_, thereby validating the model.

Figure 5 shows that the coatings from experiments 3 and 8 exhibit the highest hardness values among all samples. Interestingly, these coatings also feature the highest P contents (see Table 2). The influence of complexing agents on the P content has been reported elsewhere for ENP layers due to their ability for controlling the free Ni^2+^ concentration in the electrolyte which lately affects the composition of the coating [33]. However, in our case, several complexing agents are playing a role simultaneously and their effect cannot be straightforwardly deconvoluted. Complexing agents 1 and 2 can be regarded as “real” complexing agents, whereas other chemicals present in the electrolyte, like short chained organic acids, act mainly as pH buffers. In double-complexed systems, it has been reported that higher ratios of complexing agents can lead to higher P percentages in the NiP layers [34]. Experiment 3 had a (complexing 1/complexing 2) molar ratio of 0.236, whereas that for experiment 8 was of 0.083. Besides differences in the concentration of Ni (II) and complexing agents, both systems exhibited similar free Ni^2+^ ions concentration. Namely, free Ni^2+^ concentration was 6.8 × 10^−5^ and 3.8 × 10^−5^ for experiments 3 and 8, respectively. Accordingly, experiment 8 yielded coatings with lower P content and enhanced crystallinity (Figure 2) in comparison with experiment 3, whose coatings featured higher P content and were mechanically harder (Figure 5).

#### 3.1.4. Wear Resistance

**Figure 6:**
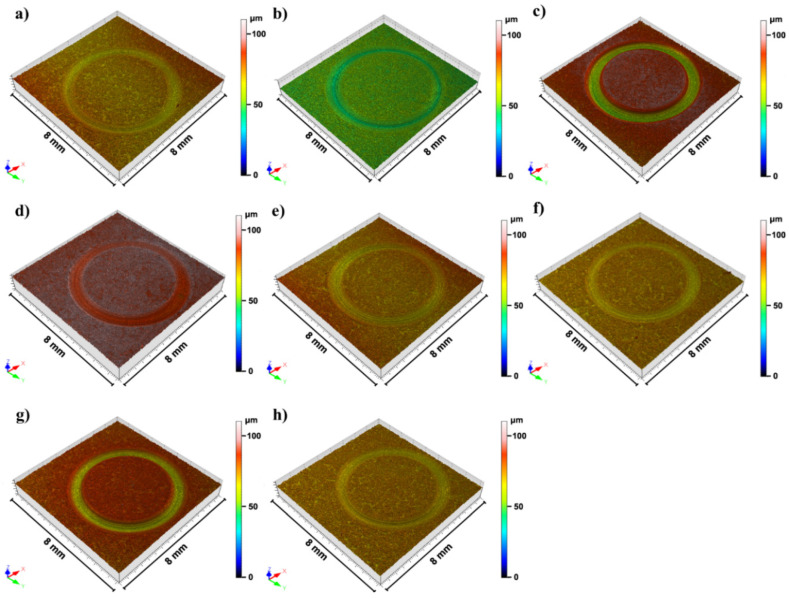
Confocal microscopy images of 3D wear tracks of coatings obtained from experiments 1–8 (**a**–**h**) and subject to wear tests.

Evaluation of the mechanical performance of ENP coatings was completed with the analysis of their wear resistance. Figure 6 gathers the 3D images of worn surfaces from NiP coatings subject to wear tests. As can be inferred from Figure 6c,g, coatings from experiments 3 and 7 displayed the highest worn volumes among all samples. For the sake of comparison, the worn values, together with the P content, thickness, hardness, and corrosion rates are listed in Table 4.

Upon looking at Figure 1d, it was concluded that temperature, and the concentrations of complexing agent 2 and reducing agent were the most influential parameters for hardness. According to this, the optimal configuration of parameters was A+B−C−D+E−F−G−. The prediction equation rendered a worn volume value of 0.045 × 10^−3^ mm^3^. The confirmation experiment analysis demonstrated that the resulting coating was characterized by a soft wear track. The worn volume determined from the 3D image was 0.045 × 10^−3^ mm^3^, again confirming the model. Smoothness and the characteristic cauliflower-like microstructure of electroless nickel coatings greatly account for their good performance in wear, which is due to the self-lubricant character of NiP coatings [35]. The conditions optimized for maximum wear resistance simultaneously promoted a higher degree of crystallinity of the coatings, which exhibited crystallite sizes in the range of 5–10 nm. The presence of small crystallites prevent movement of dislocations and other deformation mechanisms involved in plastic deformation due to the higher number of atoms placed at grain boundaries and triple junctions [9,36].

#### 3.1.5. Corrosion Resistance

**Figure 7:**
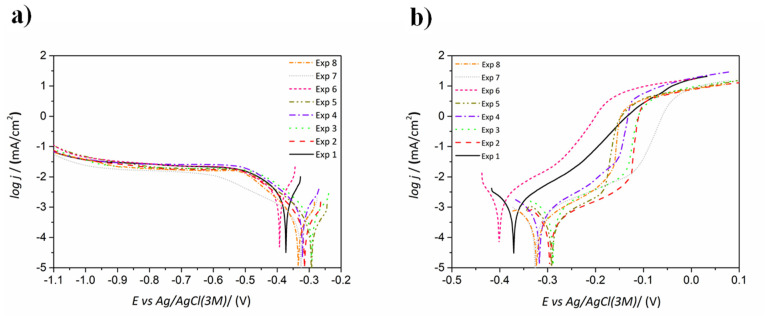
(**a**) Cathodic and (**b**) anodic branches of the polarization curves for the various ENP coatings obtained through experiments 1–8.

Corrosion resistance optimization was based on the lower-the-better criteria. The cathodic and anodic polarization curves of the different coatings are displayed in Figure 7. Significant variations were observed in the corrosion potential (E_corr_) values, with differences higher than 100 mV, suggesting the possibility to prepare coatings with variable noble character depending on the experimental conditions. Nevertheless, E_corr_ values do not give by itself information about the kinetics of the corrosion phenomena and, therefore, the corrosion rate obtained from i_corr_ by Tafel extrapolation is regarded as a better indicator [37]. In this sense, coatings obtained from experiments 2, 5 and 8 showed the lowest corrosion rate among all the samples (Table 4). The cathodic branches indicate that their E_corr_ values are in the order of experiments 8 < 2 < 5. 

Overall, the cathodic branches shown in Figure 7a indicate a very similar behavior in all samples except for the coating derived from experiment 7. In this case, an extended transition zone in the region from −0.35 to −0.70 V vs. Ag/AgCl was observed before the diffusion-controlled region. Meanwhile, the other samples all showed a diffusion-controlled region from −0.5 V vs. Ag/AgCl. At potentials more negative than −1.0 V vs. Ag/AgCl, a change in the slope of the cathodic branch is observed, indicating the beginning of hydrogen evolution as described elsewhere [38].

The anodic branches indicate that coatings resulting from experiments 2 and 5 are among those with higher corrosion resistance (see Figure 7b). In particular, coatings from experiments 2 and 5 showed more positive E_corr_ values than the coating from experiment 8, although all of them exhibit similar anodic slopes giving rise to comparable corrosion rates (see Table 4). In the three cases, the curves exhibited a pseudo-passive region for values of +150 mV vs. E_corr_. The sample from experiment 7 displayed a similar behavior, although it showed a slightly higher corrosion rate. Experiments 2, 5 and 8 corresponded to coatings with high crystallinity (Figure 2), characterized by the occurrence of the (200) plane and the (111) preferred orientation of the fcc nickel phase. High corrosion resistance has already been reported in aerated NaCl media for nanocrystalline NiP coatings irrespective of the P content, with corrosion rates comparable to those obtained in this study for the best performing coatings [38]. Except for experiment 7 derived coating, samples with low P contents (<4 wt.% P) showed lower corrosion rates, which is directly linked with their crystalline structure. The transition from nanocrystalline to mixed nanocrystalline–amorphous structure corresponds to coatings with medium wt.% P and negatively affects the corrosion performance. This is due to the presence of smaller crystals which create more active sites prone to corrosion attack through grain and nodule boundaries. Moreover, P enrichment at the outer coatings’ surface can occur under anodic polarization conditions, which is caused by preferential nickel dissolution according to previous studies [14,39,40]. Yet, this effect was not observed in our case.

According to Figure 1e, the analysis of the most influential parameters revealed that higher pH, lower stirring rate and higher reducing agent concentration led to coatings characterized by lower corrosion rates. Complexing agents and stabilizers present in ENP baths have been described as corrosion performance modifiers [41,42]. However, the concentration of the complexing agents had little influence on the corrosion performance of the studied NiP coatings according to the S/N ratio values in Figure 1e.

**Figure 8:**
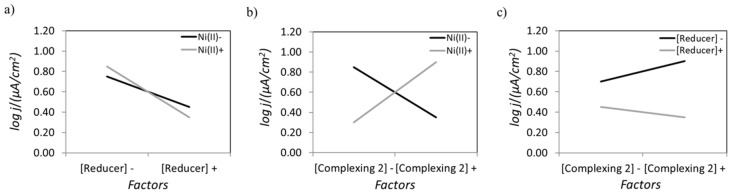
Interaction plots for some of the factors considered in the L_8_ matrix for (**a**) Ni (II) and reducing agent, (**b**) Ni (II) and complexing agent 2, and (**c**) reducing agent and complexing agent 2 concentrations.

On the other hand, the notorious influence of the stirring rate on the corrosion resistance of the coatings was somewhat unforeseen. Upon analyzing the interactions between factors, Ni (II) and complexing agent 2 concentrations showed a strong interaction with regard to the corrosion rate as suggested by the x-shaped plot depicted in Figure 8b, which indicates that the stirring effect could be poisoned by this interaction. Moreover, the interaction between factors A and B (i.e., concentrations of Ni (II) and H_2_PO_2_^−^), although present, was not considered significant according to common knowledge in electroless plating (Figure 8a).

In order to elucidate the real contribution of the interaction between the concentrations of Ni (II) (factor 1) and complexing agent 2 (factor 4), a dedicated Taguchi study was conducted using an L4 matrix, which allowed the study of two factors at two different levels in a separate manner, including the effect of their interaction on the corrosion performance (Table 5).

**Figure 9:**
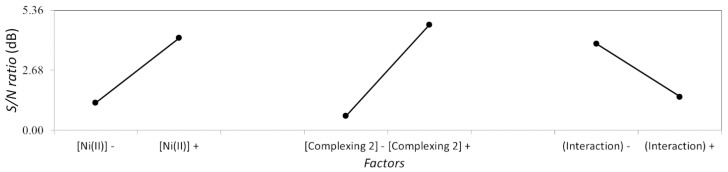
Main effect plot for a dedicated Taguchi design to determine the influence of Ni (II) and complexing agent 2 concentrations on the corrosion performance of ENP coatings.

The effect plot of the different factors, and their corresponding interaction, evaluated in the dedicated Taguchi study can be shown in Figure 9.

**Figure 10:**
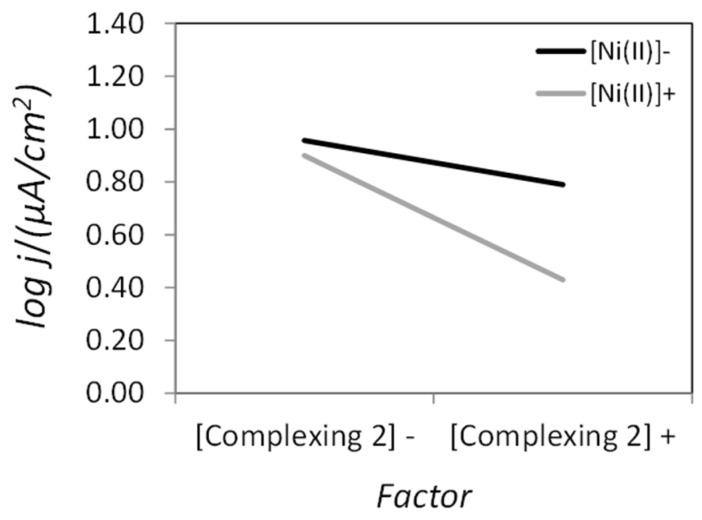
Interaction plot for a dedicated L_4_ matrix to determine the influence of Ni (II) and complexing agent concentrations on the corrosion performance of the ENP coatings.

The detailed analysis of this interaction revealed that it was indeed not significant (Figure 10), thus validating our previous outcome that stirring was key to achieve the lowest corrosion rate.

Once the effect of all factors was confirmed, the prediction equation for corrosion resistance was defined as A+B+C−D+E+F−G+, yielding a theoretical value for corrosion rate of 0.3 µA/cm^2^. A confirmation experiment conducted under the optimal parameters sequence resulted in coatings with a corrosion rate of 0.25 ± 0.05 µA/cm^2^, confirming the predicted value derived from the model. This result also confirms the importance of stirring in electroless nickel plating. It is advisable to stir the solution while plating, in order to avoid hydrogen pitting and the occurrence of patterns, although too vigorous agitation can negatively affect the plating process [43]. Moderate stirring rates are therefore recommended to secure higher diffusion rates, conveyed by convection, in order to facilitate the arrival of fresh reactants to the substrate through the diffusion layer. On the other hand, higher agitation regimes can lead to NiP coatings characterized by increased corrosion rates in chloride media [44]. Suitable agitation promotes, in acid electrolytes, the increase in pH in the diffusion layer which, in turn, facilitates the incorporation of low amounts of P in the layers as well as the growth of more compact and smoother coatings [7]. As a result, compact NiP layers with fewer defects are obtained, which ultimately causes a better response in electrochemical corrosion tests in chloride media.

### 3.2. Multivariate Optimization

The design of novel coatings is typically aimed at enhancing a given property without considering the effect that the experimental parameters might have on other properties. In this study, the most favorable set of factors have been independently determined following a univariate optimization approach in the preceding section, which is summarized in (Table 6).

On the other hand, in multivariate optimization, more than one feature is considered in the same optimization run, thus allowing for a more realistic coating design. This is of utmost importance in real world applications, where coatings are exposed to more than one degradation mechanism (i.e., wear-corrosion, fatigue-corrosion, etc.) during their service life [45,46,47]. Hence, multivariate optimization was run for all the features that were considered separately in the previous univariate optimization. 

**Figure 11:**
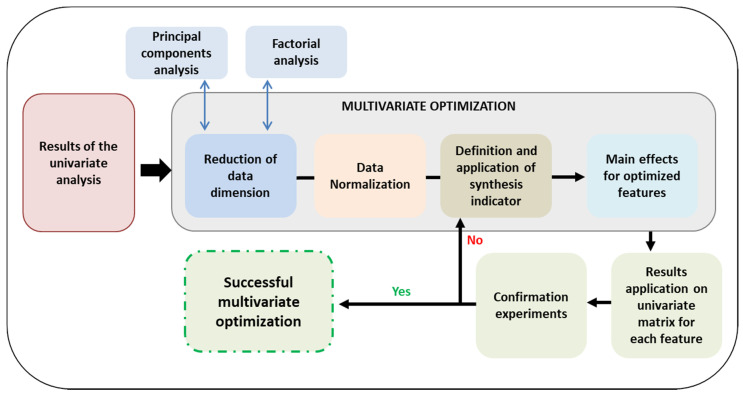
Multivariate optimization workflow used in this study.

Several approaches for multivariate optimization based on the Taguchi methodology exist such as grey relational analysis, genetic algorithms, etc. [20,48,49]. The multivariate optimization workflow used in this study is shown in Figure 11.

The relation between different factors should be first evaluated to check whether they can be expressed as a lineal combination, which permits to reduce the dimension of the original matrix. Several methods can be used for reduction of data dimensionality. Of them, the calculation of correlation coefficient was selected in this study. The correlation coefficient r is given by Equation (1), where n corresponds to the number of samples, and x_i_ and y_i_ to the values of the different factors involved in the comparison.

(1) $$r=\;\frac{n\cdot (\sum_{i=1}^{n}x_{i}\cdot y_{i})-\left(\sum_{i=1}^{n}x_{i}\right)\cdot \left(\sum_{i=1}^{n}y_{i}\right)}{\sqrt{n\left(\sum_{i=1}^{n}x_{i}^{2}\right)-{\left(\sum_{i=1}^{n}x_{i}\right)}^{2}}\cdot \sqrt{n\left(\sum_{i=1}^{n}y_{i}^{2}\right)-{\left(\sum_{i=1}^{n}y_{i}\right)}^{2}}}$$

An r = 0 means that there is not a lineal correlation between factors, r = 1 means perfect positive correlation between factors, and 0 < r < 1 indicates a certain degree of lineal correlation. The same is true for negative correlations, for which r = −1. Using the values shown in Table 4, and applying Equation (1), the resulting correlation matrix is (Equation (2)):

(2) $$r=\left(\begin{array}{cc} \begin{array}{ccc} r_{11} & r_{12} & r_{13}\\ r_{21} & r_{22} & r_{23}\\ r_{31} & r_{32} & r_{33} \end{array} & \begin{array}{ccc} r_{14} & r_{15} & r_{16}\\ r_{24} & r_{25} & r_{26}\\ r_{34} & r_{35} & r_{36} \end{array}\\ \begin{array}{ccc} r_{41} & r_{42} & r_{43}\\ r_{51} & r_{52} & r_{53}\\ r_{61} & r_{62} & r_{63} \end{array} & \begin{array}{ccc} r_{44} & r_{45} & r_{46}\\ r_{54} & r_{55} & r_{56}\\ r_{64} & r_{65} & r_{66} \end{array} \end{array}\right)=\left(\begin{array}{cc} \begin{array}{ccc} 1 & 0.634 & 0.281\\ 0.634 & 1 & 0.364\\ 0.281 & 0.364 & 1 \end{array} & \begin{array}{ccc} 0.670 & 0.495 & 0.442\\ 0.201 & 0.741 & 0.055\\ 0.410 & 0.211 & 0.302 \end{array}\\ \begin{array}{ccc} 0.670 & 0.201 & 0.410\\ 0.495 & 0.741 & 0.211\\ 0.442 & 0.055 & 0.302 \end{array} & \begin{array}{ccc} 1 & 0.489 & 0.480\\ 0.489 & 1 & 0.232\\ 0.480 & 0.232 & 1 \end{array} \end{array}\right)$$

The matrix depicted in Equation (2) shows that the highest value for the correlation coefficient among the different factors correspond to position r_25_ = 0.741 for thickness and worn volume features. Yet, although there is apparently some degree of correlation, the value does not support the existence of a lineal combination. Thus, it was not possible to reduce data dimensionality as there were no lineal combinations of variables.

According to the procedure described in Figure 11, data resulting from the univariate optimization were transformed into a normal distribution using the Zscore, as shown in Equation (3), where x is the value of each feature in the different experiments, µ is the average of all the measurements for that feature, and σ the standard deviation.

(3) $$Z=\;\frac{\left(x-\mu \right)}{\sigma }$$

A synthesis indicator (SI) was defined for the different features depending on the specific weight a definite feature would have on the overall response. Thus, according to this research, minimum wear volume and lowest corrosion resistance were the features with the highest impact in the response derived from the multivariate optimization, followed by the P content, thickness and hardness of the coatings. As the optimization of wear and corrosion resistance fell within the lower-the-better category, the sign of these features was negative. The SI values for the different properties are listed in Table 7.

By applying the SI to the obtained values, it was possible to elucidate the main effects on wear and corrosion resistance in an analogous manner to the Taguchi methodology used in the univariate optimization, but considering the two properties simultaneously. Optimal factors were achieved in positions for which the overall effect is maximized. Table 8 shows the main effect values, their ranking within the factors considered, and the optimal level (+, −) for each factor. 

Table 8 demonstrates that the most influential parameters to secure coatings with the lowest wear volume and highest corrosion resistance were pH, complexing agent 2 concentration, stirring and temperature, followed by Ni (II), reducing agent and complexing agent 1 concentrations. Thus, the optimized factors arrangement for multivariate analysis was A+B−C−D+E+F−G+. In order to obtain the prediction equation for each individual feature, the results were brought back into the univariate Taguchi matrix, where the new configuration of factors was implemented. The final projected values for each feature are shown in Table 9.

According to the predicted values, lower phosphorus content and lower corrosion rates could be achieved through a multivariate optimization, thus improving the results achieved in the univariate optimization (cf. Table 5). It should be pointed out that a different arrangement of factors was obtained compared to the univariate optimization, thus demonstrating the suitability of the multivariate approach for the optimization of this kind of multifactorial systems.

#### Results of the Confirmation Tests

**Figure 12:**
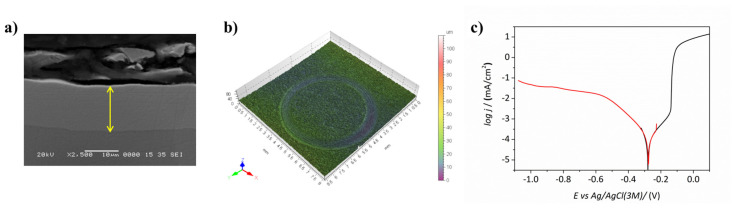
Confirmation test results for (**a**) thickness, (**b**) wear and (**c**) corrosion performance of coatings obtained from multivariate optimization.

P content, thickness, hardness, wear and corrosion resistance were evaluated on coatings obtained from the formulation and working parameters derived from the multivariate optimization and shown in Table 7. Phosphorous content evaluated by EDX amounted to 2.2 ± 0.2 wt.%, thereby confirming the predicted value. Mean coating thickness was 12.1 ± 0.4 µm (Figure 12a), which was slightly higher than the projected value, but in the range of the model considering the standard deviation of the measurement.

Hardness measurements yielded a value of 851 ± 27 Hv_0.1_, which was again in full agreement with the prediction. The worn volume determined after a tribological test was 0.068 ± 0.010 × 10^−3^ mm^3^ (Figure 12b). This value was slightly higher than the predicted value but of the same order of magnitude, and it was also higher than the best value achieved in the univariate optimization (cf. Table 5). Note that this is a global optimization and therefore some features may not reach their optimal values.

Finally, the corrosion resistance of the coatings produced for the confirmation test was assessed by electrochemical techniques. The polarization curve (cathodic plus anodic branches) of the coating obtained in the confirmation experiment is shown in Figure 12c. The corrosion rate was 0.070 ± 0.02 µA/cm^2^, which was moderately higher than the 0.020 µA/cm^2^ value predicted by the model. Nevertheless, it is worth pointing out that the value obtained after the multivariate optimization is one order of magnitude lower than the values obtained in the univariate optimization (cf. Table 4). Despite the fact that corrosion rate was not the feature with the highest specific weight in the optimization run, the experimental setup derived from the multivariate optimization had a positive effect on the corrosion performance of the coatings. In particular, higher stirring rate and lower plating rate (favored by a lower temperature) boosted the corrosion resistance, as observed in the univariate optimization (see Table 5). Low plating rate allows the growth of more compact electroless nickel coatings with lesser defects, thereby improving the corrosion performance of the NiP coatings. On the other hand, higher pH promotes the incorporation of lower amounts of P in the nickel matrix, which is beneficial for the mechanical properties. 

Table 10 summarizes the predicted and experimental results obtained from the multivariate optimization. The experimental values match very well with the predicted values, validating the methodology proposed for the optimization of complex electroless nickel formulations. Remarkably, the optimized solution renders NiP coatings with three times lower corrosion rates than the value obtained in the univariate optimization, without compromising hardness and wear performance.

## 4. Conclusions

The Taguchi method has been adopted for the optimization of a complex multicomponent electroless nickel formulation. In this study, two approaches have been employed for optimizing different features or properties, first individually (univariate approach) and later globally (multivariate approach). In either case, it has been possible to obtain valuable information from a reduced number of experiments. The P content, thickness, hardness, wear volume and corrosion resistance were chosen as the optimizable features. From the compositional point of view, the role of complexing agent 2 in modulating hardness and wear resistance of the coatings needs to be highlighted. On the other hand, pH and stirring rate have been determined as the most influential parameters for achieving coatings with low corrosion rates. Moreover, lower operation temperature favors the growth of more compact and defect-free NiP coatings.

As opposed to univariate optimization, multivariate optimization allows improving more than one feature at the same time. In this study, wear and corrosion resistance were selected as features with the highest specific weight in the multivariate optimization response. The optimal experimental setup derived from the multivariate optimization rendered a configuration of factors which was different from any of the combinations obtained in the univariate optimization. Moreover, it has been possible to obtain coatings exhibiting three times lower corrosion rates in comparison to the best result achieved in the univariate optimization stage for this feature. Surprisingly, corrosion resistance was not the feature with the highest specific weight on the overall response but was best optimized under the given set of factors. 

Accurate design of experiments is as a powerful tool for the optimization of complex electroless nickel electrolytes, providing useful information with a limited number of trials, making this methodology both time and cost effective. The proposed univariate and multivariate optimization methodologies could be extrapolated to other complex systems in which bath components (and their concentrations) and experimental conditions play important roles on the performance of the coatings.

## Figures and Tables

**Table 1:** Experimental matrix derived from L_8_ orthogonal array (OA).

Factors	A	B	C Interaction A-B	D	E Interaction A-D	F Interaction B-D	G
Experiment	Ni (II) g/L	Reducer g/L	Complexing 1 g/L	Complexing 2 g/L	Stirring rpm	T °C	pH
1	4.8	20.0	1.0	7.0	100	75	5.2
2	4.8	20.0	1.0	12.0	250	90	6.5
3	4.8	30.0	3.0	7.0	100	90	6.5
4	4.8	30.0	3.0	12.0	250	75	5.2
5	6.5	20.0	3.0	7.0	250	75	6.5
6	6.5	20.0	3.0	12.0	100	90	5.2
7	6.5	30.0	1.0	7.0	250	90	5.2
8	6.5	30.0	1.0	12.0	100	75	6.5

**Table 2:** Phosphorous content in the coatings obtained from L_8_ experiments. “S.D.” stands for standard deviation.

Experiment	1	2	3	4	5	6	7	8
P content (wt.%)	7.0	3.2	7.8	6.4	2.2	6.7	8.5	4.0
S.D.	0.1	0.2	0.1	0.1	0.3	0.1	0.3	0.1

**Table 3:** Mean effect plot for S/N ratios for P content in the ENP layers obtained following the lower-the-better optimization approach.

Factor	[Ni (II)]	[Reducer]	[Complexing 1]	[Complexing 2]	Stirring	T	pH
−	−15.3	−12.6	−14.4	−15.0	−15.8	−13.0	−17.0
+	−13.5	−16.2	−14.3	−13.7	−12.9	−15.8	−11.7
Difference	1.8	3.6	0.1	1.3	2.9	2.8	5.4
Rank	5	2	7	6	3	4	1

**Table 4:** Summary of the values of the properties of the NiP coatings obtained from the eight experiment setups.

L_8_	P wt.%	Thickness µm	Hardness Hv_0.1_	Worn Volume (×10^−3^) mm^3^	Corrosion Rate µA/cm^2^
1	7.1 ± 0.1	11.6 ± 0.1	714 ± 32	0.049 ± 0.005	1.20 ± 0.04
2	3.2 ± 0.2	16.5 ± 0.3	853 ± 23	0.068 ± 0.012	0.30 ± 0.01
3	7.8 ± 0.1	24.4 ± 1.6	860 ± 7	0.230 ± 0.085	0.50 ± 0.02
4	6.4 ± 0.1	13.1 ± 0.3	792 ± 7	0.051 ± 0.003	0.40 ± 0.01
5	2.2 ± 0.3	11.1 ± 0.2	788 ± 10	0.050 ± 0.004	0.20 ± 0.01
6	6.7 ± 0.1	20.3 ± 0.3	834 ± 5	0.045 ± 0.008	1.50 ± 0.06
7	8.5 ± 0.3	25.6 ± 1.0	777 ± 28	0.150 ± 0.021	0.40 ± 0.03
8	4.0 ± 0.1	15.4 ± 0.4	873 ± 31	0.045 ± 0.005	0.30 ± 0.02

**Table 5:** Experimental matrix derived from dedicated L_4_ OA.

Factors and Experiments	A Ni (II) g/L	B Reducer g/L	Interaction A-B
1	4.8	7	-
2	4.8	12	-
3	6.5	7	-
4	6.5	12	-

**Table 6:** Summary of the univariate optimization results for the features or properties considered in this study.

Feature	Units	Equation	Predicted	Experimental
P content	wt.%	A+B−C+D+E+F−G+	3.3	3.1 ± 0.2
Thickness	µm	A+B+C−D−E−F+G-	24.1	24.3 ± 1.6
Hardness	Hv_0.1_	A+B+C+D+E−F+G+	870	863 ± 27
Worn volume	(×10^−3^) mm^3^	A+B−C−D+E+F−G−	0.045	0.045 ± 0.005
Corrosion rate	µA/cm^2^	A+B+C−D+E+F−G+	0.30	0.25 ± 0.03

**Table 7:** Synthesis indicator (SI) values employed in the multivariate optimization.

SI	P	Thickness	Hardness	Wear	Corrosion
value	−0.2	0.1	0.1	−0.35	−0.25

**Table 8:** Main effect chart on wear and corrosion resistance of ENP coatings for multivariate optimization.

Factor	[Ni (II)]	[Reducer]	[Complexing 1]	[Complexing 2]	Stirring	T	pH
−	−0.131	0.080	0.057	−0.251	−0.204	0.186	−0.258
+	0.131	−0.080	−0.057	0.251	0.204	−0.186	0.258
Main effect	0.263	0.160	0.115	0.502	0.408	0.372	0.516
Rank	5	6	7	2	3	4	1
Optimal level	+	−	−	+	+	−	+

**Table 9:** Predicted values for all the properties subject to multivariate optimization.

Feature	Units	Predicted
P content	wt.%	2.2
Thickness	µm	10.8
Hardness	Hv_0.1_	842
Worn volume	(×10^−3^) mm^3^	0.045
Corrosion rate	µA/cm^2^	0.02

**Table 10:** Predicted and experimental values of ENP coatings subject to multivariate optimization.

Feature	Units	Predicted	Experimental
P content	wt.%	2.2	2.2 ± 0.2
Thickness	µm	10.8	12.1 ± 0.4
Hardness	Hv_0.1_	842	851 ± 27
Wear	(×10^−3^ mm^3^)	0.045	0.068 ± 0.010
Corrosion	(µA/cm^2^)	0.02	0.07 ± 0.02

## Data Availability

The data presented in this study are available on request from the corresponding author.
